# The Diagnosis of Internal Secretory Disorders

**Published:** 1916-06

**Authors:** Henry R. Harrower

**Affiliations:** M. D., F. R. S. M. (Lond.), Los Angeles, California


TEXAS MEDICAL JOURNAL

Dr. F. E. Daniel, Founder. Established July, 1885

MRS. F. E. DANIEL, - Publisher and Managing Editor

Published Monthly.—Subscription, $1.00 a Year.

VOL. XXXI AUSTIN, JUNE, 1916 No. 12

The publisher is not responsible for views of contributors

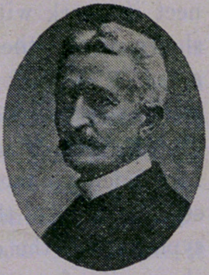

### Original Articles.

### The Diagnosis of the Internal Secretory Disorders.

BY HENRY R. HARROWER, M. D., F. R. S. M. (LOND.), LOS ANGELES, CALIFORNIA.

### II.

### THE DETECTION OF THE MINOR THYROID DYSCRASIAS.

A full appreciation of the clinical importance of the disorders of the thyroid gland presupposes a knowledge of the essential rôle that it plays in the regulation of the functions of the body. Truly it is a most wonderful organ, and it has been called very aptly the keystone of the endocrinous arch. As such it is the most important single factor in the direction of the intricate workings of metabolism, for it has been well affirmed that the thyroid governs growth and development--physical and mental, controls the breaking down of certain food materials, particularly albumen, and regulates the complex chemical processes by means of which the cellular wastes are disposed of. As a consequence of this, many different physiological manifestations are intimately bound up with the work of the thyroid and its functional disorders, no matter how slight, are immediately reflected in such functions as heat regulation, muscular efficiency, peristalsis, urinary excretion, especially the elimination of nitrogen, menstruation and other activities of the gonads, both in the male as well as the female, the different features of mental capacity, hematopoiesis, nutrition, especially of the skin and its appendages, as well as the development of features, form and functions generally.

The thyroid hormone also has much to do with the powers of the body to resist disease. Sajous was among the earliest to connect its work with the production of immunity, and it has been shown to be the most important of the numerous detoxicating agencies at work in the body. This last makes the thyroid especially susceptible to the toxemias associated with the infectious diseases and the infections, in fact the chief causes of thyroid insufficiency—the minor and by far the most frequent form especially—are the infectious diseases, principally tuberculosis, syphilis and the exanthemata and, of course, typhoid, diphtheria, rheumatic fever, influenza, erysipelas and many other acute or chronic infections.

The predominating influence of the thyroid upon the functions of the gonads renders it peculiarly sensitive to variations in the sexual life, especially in women; and emotion or sexual excess, as well as the toxic and functional disorders associated with pregnancy are frequent causes of the slighter forms of thyroid insufficiency.

The intimate relation of the thyroid to metabolism, particularly that of proteids, makes it react to that unfortunately all too common etiologic factor in so many disorders—overfeeding, and this is particularly true of a diet in which meat forms a generous part. Intoxications of all kinds—intestinal, alcoholic, drug and those due to amebae in the mouth or tonsils and to intestinal and other parasites—are not infrequent exciting causes of a breakdown in the thyroid function.

With the foregoing suggestions in mind, coupled with the fundamental physiological fact that the thyroid is as important a factor as any in the detoxicating and immunizing processes of the body, it will be clear that the detection of a minor functional disorder of this gland may be of much more service than merely to direct attention to the measures necessary to reinforce the work of the lagging gland. A much more important thing will have been accomplished if, in addition to this, the underlying causative element is laid bare and steps taken to eradicate it or to nullify its influence. Very, very often the proper adjuvant treatment of thyroid inadequacy—the treatment of its cause as well as its results—is made possible by applying our increased knowledge in the right way. Hence we would naturally supplement thyroid medication with emetine where alveolar or tonsilar amebiasis is present, whether the infection has made itself prominent or not. Bacterial vaccines would be administered where there is a definite underlying bacterial origin autogenous vaccines where cultures may be made easily, or, better still, mixed stock vaccines, especially where it is difficult to locate the nidus of infection and secure a culture. Again, systemic alkalinization is very much in order where acidosis and toxemia are prominent; in fact, in almost every case of chronic benign thyroid insufficiency, generous and judiciously timed doses of sodium bicarbonate will make the response to thyroid therapy much more satisfactory. Where intestinal concretions are palpable and stasis is obvious, eliminant and lubricating remedies facilitate the best results by removing the wastes which not merely aggravate the manifestations of thyroid disorder, but hinder every function of the body.

For these reasons, then, I rarely employ thyroid medication without some associated treatment, and am confident that many a failure in this line of therapeutic effort is due to the omission of the necessary adjunct measures; for many a time it will be found that a certain method of treatment is rendered much more efficient by the addition of thyroid (say, one-fourth or one-half grain three times a day), while the reverse is equally true—thyroid therapy is enhanced by combining with it other suitable treatment the need for which is too often overlooked.

Before we pass from the consideration of the underlying basis of thyroid disorder a word of emphasis may be advantageously placed right here. Has it ever occurred to the reader that one of the commonest features of the heavy eater or drinker is obesity, and that this is also a common and quite constant feature of hypothyroidism? Why should the thyroid glands of those who are wont to abuse their bodies be so immune to the influence of the very factors which most commonly disturb their function? They are not, and an almost constant feature of such individuals is hypothyroidism manifested in not a few disorders other than the one just mentioned, of which we shall shortly learn.

All these exciting factors, and others quite similar to them, are clearly much more effectual disturbers of the chemical routine of the thyroid gland when the individual has an unstable thyroid mechanism to start with; and as with the major thyroid diseases, about which we shall have more to say later, *heredity* is the one great foundation upon which a susceptibility to thyroid dyscrasia is built. This is the great predisposing cause, while the toxemias and other circumstances previously mentioned are the exciting causes.

Many conditions combine to favor the production of congenitally subthyroidic children, most important among which are various degrees of the same disorder in the parents and especially the mother. Transmitted tendencies toward tuberculosis, malnutrition and other congenital ills to which the flesh is all too often heir, not forgetting inherited syphilis—a most potent cause of every kind of functional and organic diseases of the glands of internal secretion—are almost invariably associated with thyroid instability, and by this is meant, not necessarily definite thyroid disease of varying degree, but an inherent weakness of the gland which permits it to succumb to the first serious stress that may be put upon it.

Usually this extra strain may be the result of some of the common infectious diseases of childhood (incidentally children with this unstable condition are just the ones who "catch everything") or it may not appear until puberty, at which period many a thyroid insufficiency of more or less permanency first makes itself manifest.

Other factors which favor the hereditary subthyroidism in children are toxemias during pregnancy and labor prior to their birth. I have frequently found a connecting thread between such conditions and the complex endocrinous disorders which I am so often called to see, and it would be interesting to see a report of the thyroid findings in a goodly number of individuals whose advent into the world was the occasion of eclampsia, some figures which I do not believe have yet crept into medical literature. Important among the other predisposing hereditary causes of thyroid instability are unduly frequent childbearing, prolonged lactation and other physical strains upon the system of the mother during pregnancy. Acute infectious diseases of the mother may cause this unfortunate tendency in her offspring; though it should be said with emphasis that these conditions just mentioned do not necessarily spell thyroid inadequacy in the child and, of course, a constitution may be acquired after birth and in spite of a poor heredity.

Perhaps additional emphasis should be given to the importance of syphilis as a predisposing as well as an exciting factor in this class of cases. We are taught to consider syphilis as a prospective cause of every obscure and difficult condition and to presume its presence until it is definitely ruled out. This is the correct way to look at the subject, though it is not the usual way. The advent of the Wassermann test and its standardization and control by other procedures has made it possible to know with definiteness whether the syphilitic factor is present or not, and every insidious case of this character should have the benefit of the Wassermann test at least of the blood, and not infrequently of the spinal fluid also.

Syphilis is the most insidious of all diseases and is most protean in its manifestations, and Dr. Lewellys F. Barker, of Johns Hopkins University, was right when he recently told the New York Academy of Medicine that "the more my experience grows, the more I am inclined to take as a diagnostic aphorism, 'When in doubt have a Wassermann test made; when not in doubt still have a Wassermann test made.'" And in no class of disorders is this more truly applicable than the obscure and insidious, just like the obvious and organic, diseases of the glands of internal secretion.

Having attempted to direct attention to the numerous contributory causes of thyroid dyscrasias, as well as to the factors which are likely to precipitate slight or well marked thyroid insufficiency, we can now more intelligently proceed to consider the clinical results of this condition, and to the study of how to detect the usual and unusual symptoms of this common disorder.

---

[Editor's Note.—Thyroid dyscrasias are by far the most common of all the endocrinous disorders and the clinical findings more numerous and complicated, hence the consideration of this subject constitutes the largest unit in this series. It has been thought advisable to divide this article, and the remaining half will appear in our issue for July.]

---

Next month a continuation of the article on "The Detection of the Minor Thyroid Dyscrasias." Even more interesting than the above!

